# Changes in Physical Activity and Depressive Symptoms During COVID-19 Lockdown: United States Adult Age Groups

**DOI:** 10.3389/fpsyg.2022.769930

**Published:** 2022-02-17

**Authors:** Amy Chan Hyung Kim, James Du, Damon P. S. Andrew

**Affiliations:** Department of Sport Management, Florida State University, Tallahassee, FL, United States

**Keywords:** COVID-19, physical activity, health behavior, mental health, depressive symptom

## Abstract

This study investigates: (1) the changes in three major health-related factors—physical activity, non-physical-activity health behavior (i.e., diet quality, alcohol consumption, smoking, sleep quality), and depressive symptoms, and (2) how changes in physical activity were associated with changes in one’s depressive symptoms among young adults, middle-aged adults, and older adults while controlling non-physical-activity health behavior and sociodemographic characteristics among young, middle-aged, and older adults before and after the COVID-19 outbreak lockdown in the United States. A total of 695 participants completed an online questionnaire *via* MTurk, and participants were asked to recall their physical activity, depressive symptoms, and non-physical-activity health behavior status in January and May of 2020. The IPAQ-SF was used to evaluate individuals’ physical activity, while the CES-D-10 was used to assess depressive symptoms. Covariates included non-physical-activity health behavior and sociodemographic factors. A Bayesian significance testing of changes was used to examine significant changes in physical activity, non-physical-activity behavior, and depressive symptoms in each age group while Bayesian regression analysis was employed to examine how the changes in physical activity were associated with respondents’ depressive symptoms while controlling for individual NHB and sociodemographic characteristics. The results showed that the participants tended to maintain their physical activity levels after the lockdown despite significant increases in sitting time among young and older adults. Decreases in moderate physical activity frequency were associated with a higher level of depressive symptoms (*R*^2^ = 17.1%). Although young and middle-aged cohorts experienced fewer differences in depressive symptoms compared to their counterparts in the older group, we found no significant heterogeneity effects in the relationships of interest across all age groups. Considering different influences of physical activity on depressive symptoms depending on different levels of activity and ages, more randomized clinical trials with program-based intervention studies should be conducted with different physical activity programs for different age populations.

## Introduction

Since the COVID-19 outbreak, non-pharmaceutical public health interventions, such as shelter-in-place orders, social distancing, and isolation, have been implemented widely worldwide. Although quarantine is the best option to stop spreading the coronavirus (Islam et al., 2020), the collateral effects of quarantine are still not known very well (Jimenez-Pavon et al., 2020). Even though the level and timing of these restrictions varied from county to county, and state to state, almost all states of the United States except for Arkansas, Iowa, Nebraska, and North Dakota, issued stay-at-home orders, forcing people to stay home as much as they can to prevent the spread of COVID-19 since March 2020 (Elassar, 2020; Putt, 2020). This increased isolation has spurred noticeable lifestyle changes (Du et al., 2021). Among many changes, the present study focuses on three significant health-related changes: changes in physical activity (PA), non-physical-activity health behavioral patterns (NHB; i.e., diet quality, alcohol consumption, smoking, sleep behavior), and depressive symptoms (DS) among young (18–39 years old), middle-aged (40–59 years old), and older adults (60 years old and older) in the United States.

There are two different tales about the lockdown effect resulting from COVID-19. On one hand, due to the stay-at-home orders and social isolation/distancing guidelines, many opportunities to be physically active were interrupted, leading to decreased levels of PA and increased sedentary behavioral patterns, including more screen and sitting times (Hall et al., 2020; Hammami et al., 2020), poorer diet quality, increased alcohol consumption and smoking, and irregular sleep quality (Mattioli et al., 2020a,b; Patwardhan, 2020; United Nations, 2020). More time spent outdoors is well-known to associate with higher levels of PA and lower levels of sedentary behavior (Schaefer et al., 2014; Eigenschenk et al., 2019). Increased time spent indoors after the COVID-19 confinement is expected to result in decreased levels of PA among the public (Woods et al., 2020). Few empirical studies have supported this expectation for a decrease in PA levels. Lopez-Bueno et al. (2020) reported a 20% decrease in weekly PA levels among Spanish Adults, while male participants over the age of 42 without a university degree had the greatest reduction. Yamada et al. (2020) found a significant decrease in PA levels among older adults (65–84 years old) in Japan in April 2020 compared to January 2020. The social confinement due to COVID-19 may result in unhealthy behaviors in that socially isolated and lonely individuals tend to have less favorable lifestyles, including poor nutritional habits and increased risk behaviors such as smoking or alcohol consumption (Loucks et al., 2006; Grippo et al., 2007; Mattioli et al., 2020b). In regard to nutritional habits, the COVID-19 outbreak may have forced the public to buy more packaged and long-life food rather than fresh food, resulting in an unhealthy diet and a reduced intake of antioxidants. Moreover, the lack of emotional support from one’s social network, friends, and family is significantly associated with stress-driven alcohol consumption (Mattioli et al., 2020a) and an increased level of smoking among smokers, as well as increased relapses among ex-smokers (Patwardhan, 2020). Historically, disease-related quarantines lead to a high prevalence of psychological distress, anxiety and stress (DiGiovanni et al., 2003; Hawryluck et al., 2004; Lee et al., 2005). During the COVID-19 pandemic, several studies consistently reported that poorer psychological well-being and mental health was present compared to the pre-COVID-19 era due to financial-related stress from insecurity and financial losses (Purtle, 2020), social isolation (Banerjee and Rai, 2020; Golechha, 2020; Pelizza and Pupo, 2020), confusion from conflicting messages from authorities and media platforms (Pfefferbaum and North, 2020), unpredictability, uncertainty, and seriousness of the disease (Zandifar and Badrfam, 2020), high levels of fear and panic behavior such as stockpiling of resources (Mattioli et al., 2020b; Shigemura et al., 2020), or poor perceived health (Wang et al., 2020).

On the other hand, thanks to more free time resulting from work-from-home trends, people may spend more time on their leisure activities, including physical activity, gardening, or Do-It-Yourself (DIY) projects. During the lockdown, Roberts (2020) found that people with additional free time increased exercises such as walking, jogging, running, and cycling despite the shutdowns of sport facilities and sport events. However, the author noted that this increased time spent being physically active after the pandemic lockdown might be mostly by those who already partake in habitual regular exercises, recreational activities, and physical activities. The results also reported less alcohol consumption, except among heavy drinkers before the lockdown (Alcohol Change UK, 2020; CGA, 2020). In terms of sleep quality, it is expected that social isolation, disruption of daily life, greater work and family stress, excessive screen time, and stress-related fatigue resulting from the pandemic may negatively affect sleep quality (Sleep Foundation, 2020). Interestingly, several studies found that even though sleep duration was increased during the COVID-19 lockdown (Blume et al., 2020; Roberts, 2020), sleep quality was reduced (Blume et al., 2020; Fu et al., 2020; Pinto et al., 2020; Wright et al., 2020).

We identified three major research gaps in the studies of changes in PA, NHB, and DS after the COVID-19 quarantine. First, there is a lack of empirical evidence to confirm the (un)changes after the COVID-19 lockdown. Even though several scholars expected lower PA levels, poorer NHB, and more DS due to the COVID-19 confinement, these ideas were mostly experience-based commentaries without any empirical evidence (Rajkumar, 2020). Among the very few empirical studies based on COVID-19 data, Callow et al. (2020) found a negative association between levels of PA and levels of DS among adults over the age of 50 in North America. However, the study did not consider the potential existence of (un)changes before and after the COVID-19 quarantine. Second, scholars have overlooked the different population characteristics among adults—especially age—when examining the association between PA, NHB, and DS after COVID-19. For instance, Stephens (1988) found that the association between physical activity and mental health was stronger among younger adults (20–39 years old) compared to middle-aged/older adults (40 years and older). Furthermore, Fukukawa et al. (2004) found that, while there was a significant relationship between the levels of daily walking activity and DS among older adults (65–79 years old), no significant relationship was found among middle-aged adults (40–64 years old). In the same context, even though several studies identified age as a crucial factor in regard to the relationship between the levels of PA, NHB, and DS (Roberts, 2020), post-pandemic studies in this area have not considered the age effect (Lopez-Bueno et al., 2020). Lastly, there is a lack of relational studies regarding changes after the COVID-19 pandemic. That is, even though a number of studies have confirmed that individual DS is predicted by one’s PA in general, no studies investigated how changes in PA may predict changes in DS among adults after the COVID-19 pandemic.

Taken together, to fill these gaps, the aims of this study are to investigate (1) the changes in three major health-related factors (i.e., PA, NHB, and DS) and (2) how changes in PA predict changes in one’s DS while controlling NHB and personal characteristics among young adults, middle-aged adults, and older adults before and after the COVID-19 lockdown in the United States.

## Materials and Methods

### Participants and Study Design

**FIGURE 1:**
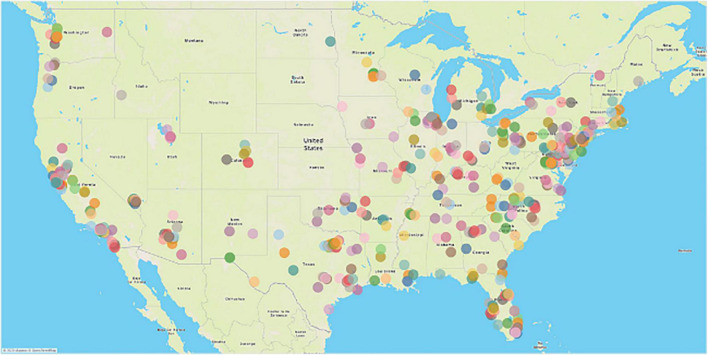
Visualization of zip code analysis of survey respondents. Color represents different zip codes in the 48 contiguous states in the United States.

Respondents were recruited *via* Amazon Mechanical Turk (MTurk), a crowd-sourcing platform where individuals complete paid tasks for various organizations. The platform has been used frequently as a data collection method in health and medical research (Ranard et al., 2014; Mortensen and Hughes, 2018). The benefits of using MTurk include higher reachability, higher reliability, and higher completion rate (Mason and Suri, 2012; Rouse, 2015; Smith et al., 2015; Hauser and Schwarz, 2016). Three different survey pools based on the different non-institutionalized, United States resident, age groups were created: young (18–39 years), middle-aged (40–59 years), and older (60 years and older) (Frayer et al., 2017). The survey was conducted in the first week of June of 2020. After obtaining consent, respondents were directed to Qualtrics to answer screening questions about the participant’s United States residency and age. We also included one attention question in the middle of the questionnaire (i.e., to continue with the survey, please select “Somewhat agree”). The current study was cross-sectional and based on a retrospective recall using a two-wave setup. The participants who passed the screening questions were directed to a questionnaire in which the participants were asked to recall their PA, DS, and NHB in January. Then, the same questions about their PA, DS, and NHB in May of 2020 were asked. The platform provides the ability to use a response validation function that alerts a respondent about questions they may have missed in each panel so that the participant can answer every question before they can proceed to the next panel of the survey. This procedural remedy eliminates missing values in the dataset. Among 865 recorded responses who passed the screening questions, a total of 170 responses (55 young, 79 middle-aged, 36 older adults), which failed to pass the attention question, were excluded. In total, 695 responses (*n*_*young*_ = 264, *n*_*middle–aged*_ = 234, and *n*_*older*_ = 197) were included for further analysis. As shown in visualization in Figure 1, survey respondents emanated from all 48 contiguous states with the exception of four states in the Midwest, including Montana, Wyoming, South Dakota, and Nebraska. Notably, COVID-19 least impacted these rural communities in the Midwest in the early period of the pandemic (Zylla and Hartman, 2020), leaving us a reasonable representative sample frame for conducting the subsequent statistical analyses. This study protocol was approved by the Institutional Review Board at Florida State University (ID: STUDY00001406).

### Measures

#### Personal Characteristics

A total of 8 items were included for measuring the sociodemographic characteristics of age, marital status, household income, education, occupation, height, weight, and zip code. We also computed an individual’s BMI using the reported height and weight.

#### Physical Activity

Self-reported PA levels of January 2020 and May 2020 were assessed using the International Physical Activity Questionnaire—Short Form (IPAQ-SF), a validated measurement tool for PA used various adult population surveys (Lee et al., 2011). The questionnaire included seven items to record the frequency and duration of four different levels of weekly physical activities—vigorous activity (e.g., aerobics or heavy lifting), moderate activity (e.g., leisure cycling or doubles tennis), walking, and sitting as of January 2020 and May 2020, respectively.

#### Non-physical-Activity Health Behavior

With acceptable validity and reliability, we used four items to evaluate one’s diet quality, including daily intakes of fruit and vegetables and weekly intakes of fast food and soft drinks (Oftedal et al., 2019). Alcohol consumption was assessed by the validated 3-item AUDIT Alcohol Consumption Questions (AUDIT-C) (Bush et al., 1998; Bradley et al., 2007; Barry et al., 2015), including questions about frequency and intensity of regular drinking and heavy drinking (e.g., “How often did you have a drink containing alcohol; How many drinks did you have on a typical day when you were drinking”). Smoking status was assessed by the validated 8-item Fagerstrom Test for Nicotine Dependence (FTND) (Heatherton et al., 1991; Hudmon et al., 2005), including frequency, amount, and dependency of smoking (e.g., “How soon after waking do you smoke your first cigarette”; “How many cigarettes a day do you smoke”; “Do you smoke even if you are sick in bed most of the day”). Sleep quality was measured by 3 items from the validated Pittsburg Sleep Quality Index (PSQI), which includes average daily hours of sleep, overall sleep quality, and sleep latency (e.g., “How would you rate your sleep quality overall”; “How many hours of actual sleep did you get at night”; “How long in minutes does it usually take you to fall asleep each night”) (Buysse et al., 1989; Carpenter and Andryknowski, 1998; Grandner et al., 2006). Overall sleep quality ranged from *1* = *very good to 4* = *very bad*. Similar to IPAQ and CES-D-10, the respondents answered the two sets of questions—(diet quality, AUDIT-C, FTND, and PSQI) for both the January 2020 and May 2020 timeframes.

#### Depressive Symptoms

The Center for Epidemiologic Studies Depression Scale (CES-D-10) was adopted to assess one’s depressive symptoms (Andresen et al., 1994). This 10-item questionnaire has been widely validated and used in studies of all age groups of adults (Noh et al., 2015; Wolanin et al., 2016; Mohebbi et al., 2018). The items reflect the feelings of the respondents (e.g., “I had trouble keeping my mind on what I was doing”; “I felt hopeful about the future”), and each item is ranged from *0* = *rarely or none of the time (less than 1 day per week)* to *3* = *most or all of the time (5–7 days per week).* The respondents answered 10 items for January 2020 and 10 items for May 2020, respectively. A compound score was generated by calculating the sum of 10 items. While a higher score indicates a higher level of DS, an individual with a score equal to or greater than ten was considered as depressed as previously recommended (Björgvinsson et al., 2013).

### Bayesian Analyses

First, for testing significant changes in PA, NHB, and DS in each age group, we employed Bayesian significance testing of changes in R using the WinBUGS package (Sturtz et al., 2005). The Bayesian approach is less sensitive to the influence associated with missing values and does not require asymptotic assumptions. It is also computationally inexpensive to generate posterior distributions based on observed changes using the Markov Chain Monte Carlo (MCMC) algorithm (Baker et al., 2019). The goal was to examine if the predicted posterior credible intervals contain the value of zero, inferring no significant changes in a parameter were likely to be observed in a given age group at the population level.

Next, a Bayesian regression analysis was employed to examine how the changes in PA was associated with the changes in respondents’ DS while controlling for individual NHB and personal characteristics. We used uniform distributions in the Bayesian significance testing of changes and employed baseline distributions of each parameter in January of 2020 as informative priors in the Bayesian regression model.

### Robustness Checks With Machine Learning

To justify the robustness of regression results, machine learning analyses, including Random Forests (RF), Support Vector Machine (SVM), and Naïve Bayes (NB), were performed in Python. Machine learning techniques provide a repertoire of promising analytical strategies to validate if significant predictors identified from the regression analysis can improve the prediction accuracy of depression as a binary mental health outcome. Consistent with recommendations from machine learning literature, we used an 80:20 split to form training and testing datasets and employed Gini decrease (GD) and information gain (IG) to determine the variable importance (Breiman, 2001; Steinwart and Christmann, 2008). Finally, three confusion matrices were generated to assess the performance of machine learning models regarding the prediction accuracy.

## Results

Descriptive statistics of respondents are displayed in Table 1, and the baseline averages of the included parameters are presented in Table 2. Roughly 60% of respondents were male, and a majority of participants were Caucasian (70.20%), well-educated (74.8% possessed a 4-year college or more advanced degrees), and lived in a typical middle-class family with annual household income ranging from $25,000 to $75,000 as of 2019. Additionally, more than 80% of the participants were employed as either a full-time or part-time employee. The pattern of demographics breakdown was mostly consistent across three age groups, except more female members (64.5%) were identified in the older adult segment.

**TABLE 1:** Descriptive statistics of sample demographic profiles.

Age groups	Parameter	Mean/Mode^†^	Frequency	Percent
				
Globalsample(*n* = 695)	Age	45.85	n/a	n/a
	Std. deviation of age	15.42	n/a	n/a
	Ethnicity	Caucasian	488	70.20%
	Gender	Male	417	60.00%
	Education	4-year college and advanced degrees	520	74.80%
	Income	$25,000 to $75,000	429	61.80%
	Job	Employed (Full-time + part-time)	570	82.00%
Young (18–39)(*n* = 264)	Age	29.78	n/a	n/a
	Std. Deviation of Age	4.08	n/a	n/a
	Ethnicity	Caucasian	161	61.00%
	Gender	Male	189	71.60%
	Education	4-year college and advanced degrees	219	82.90%
	Income	$25,000 to $75,000	159	60.30%
	Job	Employed (Full-time + part-time)	253	95.80%
Middle aged(40–59)(*n* = 234)	Age	46.74	n/a	n/a
	Std. deviation of age	4.93	n/a	n/a
	Ethnicity	Caucasian	152	65.00%
	Gender	Male	158	67.50%
	Education	4-year college and advanced degrees	193	82.40%
	Income	$25,000 to $75,000	154	65.80%
	Job	Employed (Full-time + part-time)	217	92.80%
Old (60+)(*n* = 197)	Age	66.00	n/a	n/a
	Std. deviation of age	4.50	n/a	n/a
	Ethnicity	Caucasian	175	88.80%
	Gender	Female	127	64.50%
	Education	4-year college and advanced degrees	108	54.80%
	Income	$25,000 to $75,000	116	58.90%
	Job	Employed (Full-time + part-time)	100	50.80%

*^†^means were reported for continuous variables and modes were displayed for ordinal (e.g., income) or categorical variables (e.g., gender); n/a, not applicable.*

**TABLE 2:** The significance of changes in health indicators by age groups using Bayesian *t*-tests.

Age groups	Parameter	Baseline mean in January	Parameter	Posterior		95% CI		
				Mean	SD	*p* (2-tailed)	Lower bound	Upper bound
Globalsample(*n* = 695)	VPA_D	2.85	Δ_VPA_D	−0.013	1.865	0.855	−0.152	0.126
	VPA_T	2.73	Δ_VPA_T	−0.002	2.145	0.985	−0.161	0.158
	MPA_D	3.23	Δ_MPA_D	0.045	1.897	0.536	−0.097	0.186
	MPA_T	2.59	Δ_MPA_T	−0.060	1.951	0.420	−0.205	0.086
	WPA_D	3.95	Δ_WPA_D	0.033	2.001	0.663	−0.116	0.182
	WPA_T	2.30	Δ_WPA_T	−0.084	1.932	0.251	−0.228	0.060
	SPA_T	5.26	Δ_SPA_T	0.432***	2.384	0.000	0.255	0.610
	SQ1_H	6.35	Δ_SQ1_H	0.339***	1.752	0.000	0.209	0.470
	SQ2_M	15.03	Δ_SQ2_M	2.948***	13.961	0.000	1.907	3.989
	OSQ	2.93	Δ_OSQ	0.056	0.834	0.077	−0.006	0.118
	DQ_H	5.24	Δ_DQ_H	1.827***	8.164	0.000	1.218	2.436
	DQ_UH	6.49	Δ_DQ_UH	0.449	9.586	0.217	−0.266	1.164
	Alcohol	3.40	Δ_Alcohol	−0.188***	1.107	0.000	−0.271	−0.106
	CESD_Total	11.62	Δ_CESD_Total	1.1856***	4.608	0.000	0.842	1.529
Young (18–39)(*n* = 264)	VPA_D	3.55	Δ_VPA_D	−0.129	2.050	0.308	−0.378	0.121
	VPA_T	3.02	Δ_VPA_T	0.203	2.024	0.104	−0.043	0.449
	MPA_D	3.52	Δ_MPA_D	0.140	2.032	0.263	−0.107	0.387
	MPA_T	3.03	Δ_MPA_T	−0.031	2.114	0.811	−0.288	0.226
	WPA_D	4.02	Δ_WPA_D	−0.030	1.969	0.803	−0.270	0.209
	WPA_T	2.89	Δ_WPA_T	−0.186	1.944	0.122	−0.422	0.051
	SPA_T	4.62	Δ_SPA_T	0.712***	2.619	0.000	0.394	1.031
	SQ1_H	5.97	Δ_SQ1_H	0.473***	1.804	0.000	0.254	0.693
	SQ2_M	14.30	Δ_SQ2_M	2.614**	12.889	0.001	1.046	4.182
	OSQ	2.84	Δ_OSQ	0.129*	0.955	0.029	0.013	0.245
	DQ_H	6.00	Δ_DQ_H	2.610***	9.936	0.000	1.401	3.819
	DQ_UH	7.79	Δ_DQ_UH	1.402*	11.578	0.050	0.007	2.810
	Alcohol	3.91	Δ_Alcohol	−0.393***	1.232	0.000	−0.544	−0.244
	CESD_Total	15.15	Δ_CESD_Total	0.318	4.354	0.236	−0.211	0.848
Middle aged(40–59)(*n* = 234)	VPA_D	3.09	Δ_VPA_D	−0.064	2.047	0.632	−0.329	0.201
	VPA_T	3.17	Δ_VPA_T	−0.232	2.369	0.136	−0.538	0.075
	MPA_D	3.31	Δ_MPA_D	−0.038	1.899	0.757	−0.284	0.207
	MPA_T	2.89	Δ_MPA_T	−0.138	2.120	0.321	−0.412	0.136
	WPA_D	3.71	Δ_WPA_D	0.192	2.247	0.192	−0.098	0.483
	WPA_T	2.51	Δ_WPA_T	−0.048	2.121	0.728	−0.323	0.226
	SPA_T	4.88	Δ_SPA_T	0.202	2.451	0.209	−0.115	0.519
	SQ1_H	6.21	Δ_SQ1_H	0.504***	2.089	0.000	0.234	0.774
	SQ2_M	13.48	Δ_SQ2_M	3.560***	13.180	0.000	1.855	5.265
	OSQ	2.86	Δ_OSQ	0.090	0.891	0.125	−0.026	0.205
	DQ_H	5.98	Δ_DQ_H	2.098***	9.127	0.001	0.918	3.279
	DQ_UH	7.60	Δ_DQ_UH	0.218	10.781	0.757	−1.177	1.613
	Alcohol	3.61	Δ_Alcohol	−0.137	1.232	0.091	−0.296	0.023
	CESD_Total	13.19	Δ_CESD_Total	0.915**	4.923	0.005	0.278	1.551
Old (60+)(*n* = 197)	VPA_D	1.62	Δ_VPA_D	0.203*	1.278	0.027	0.023	0.383
	VPA_T	1.84	Δ_VPA_T	−0.003	2.001	0.986	−0.285	0.280
	MPA_D	2.73	Δ_MPA_D	0.015	1.701	0.900	−0.225	0.255
	MPA_T	1.63	Δ_MPA_T	−0.005	1.459	0.963	−0.211	0.201
	WPA_D	4.13	Δ_WPA_D	−0.071	1.710	0.560	−0.313	0.170
	WPA_T	1.28	Δ_WPA_T	0.009	1.662	0.940	−0.226	0.244
	SPA_T	6.55	Δ_SPA_T	0.331*	1.896	0.015	0.063	0.598
	SQ1_H	7.04	Δ_SQ1_H	−0.036	1.056	0.637	−0.185	0.114
	SQ2_M	17.85	Δ_SQ2_M	2.670*	16.126	0.021	0.392	4.948
	OSQ	3.11	Δ_OSQ	−0.081*	0.519	0.029	−0.154	−0.008
	DQ_H	3.34	Δ_DQ_H	0.457***	1.307	0.000	0.272	0.641
	DQ_UH	3.43	Δ_DQ_UH	−0.553***	2.241	0.001	−0.870	−0.237
	Alcohol	2.49	Δ_Alcohol	0.025	0.626	0.570	−0.063	0.114
	CESD_Total	5.01	Δ_CESD_Total	2.670***	4.198	0.000	2.077	3.263

*Monte carlo sampling seed: 200,000.*

*CI, credible intervals; D, number of days per week; DQ, dietary quality (DQ_H, healthy diet; DQ_UH, unhealthy diet); H, hours of actual sleep; M, minutes to fall asleep; MPA, moderate intensity physical activity; OSQ, overall sleep quality; SPA, sitting; SQ, sleep quality (SQ1_H, total hours of sleep, SQ2, M, total minutes to fall asleep); T, total time in hours on each day; VPA, vigorous intensity physical activity; WPA, walking.*

*p < 0.05; **p < 0.01; and ***p < 0.001.

### Bayesian Analyses

#### Bayesian Significance Testing of Changes

The global results of the Bayesian significance testing of changes (see Table 2) indicated that, although slight decreases of hours in participating in PA across all three intensity levels were observed (e.g., Δ_VPA_T = −0.002, Δ_VPA_T = −0.060, and Δ_VPA_T = −0.084), the changes were not statistically significant. Nevertheless, more frequent VPA was found among the older adults (Δ_VPA_D = 0.023, *p* < 0.05). This finding suggested that people across all age groups at least maintained their activity levels even after the COVID-19 lockdown despite several challenges for individuals in engaging physically active leisure. In contrast, the participants experienced higher levels of sitting time [Δ_SPA_T = 0.432, *p* < 0.001 with 95% posterior credible intervals (CI) (0.255, 0.610)] in general. Except for the middle-aged respondents, young adults [Δ_SPA_T = 0.712, *p* < 0.001, 95% CI (0.693, 0.254)] and older adults [Δ_SPA_T = 0.331, *p* < 0.05, 95% CI (0.063, 0.015)] had a significantly increased sitting time indicating an increased level of sedentary behavior during the lockdown.

A significant increase in healthy diet was found in general (Δ_DQ_H = 1.827, *p* < 0.001). While all three age groups experienced significant increases in healthy eating, young adults experienced an increased consumption of unhealthy food [Δ_DQ_UH = 1.402, *p* < 0.05, 95% CI (2.810, 0.007)] and older adults’ unhealthy diet was decreased significantly [Δ_DQ_UH = −0.553, *p* < 0.001, 95% CI (−0.237, −0.870)]. In terms of alcohol consumption, overall, there was a significant decrease [Δ_Alcohol = −0.188, *p* < 0.001, 95% CI (−0.106, −0.271)]. This may be due to the significant decrease in alcohol consumption among young adults [Δ_Alcohol = −0.393, *p* < 0.001, 95% CI (−0.244, −0.544)]. There were no significant changes in smoking in terms of the number of smokers and dependency level across all three age groups.

When it comes to sleep quality, we found a significant increased overall sleep quality among young adults [Δ_OSQ = 0.129, *p* < 0.05, 95%CI (0.245, 0.013)] but a significant decreased overall sleep quality among older adults [Δ_OSQ = −0.081, *p* < 0.05, 95% CI (−0.008, −0.154)]. This may be due to increased sleep time among young adults [Δ_SQ1_H = 0.473, *p* < 0.001, 95% CI (0.693, 0.254)]. The middle-aged respondents also slept longer [Δ_SQ1_H = 0.504, *p* < 0.001, 95% CI (0.774, 0.234)], but there was no significant change in overall sleep quality.

**FIGURE 2:**
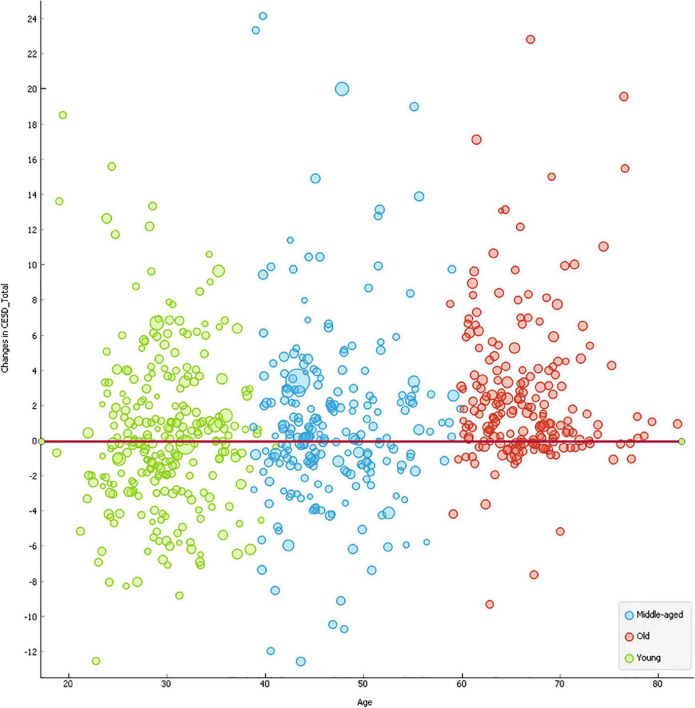
Scatterplots of changes in mental health outcomes by age. The size of the circle was indicative of the individualistic variation in BMI. The red line indicates the benchmark for zero net change in CESD between January and May.

There was no significant change in DS among the younger participants whereas the middle-aged [Δ_CESD_Total = 0.0.915, *p* < 0.01, 95% CI (1.551, 0.278)] and older participants [Δ_CESD_Total = 2.670, *p* < 0.001, 95% CI (3.263, 2.077)] had significantly higher levels of DS after the lockdown. As presented in Figure 2, the most of older adults experienced significantly increased levels of DS compared to younger and middle-aged adults.

#### Bayesian Regression Analysis

The results of Bayesian regression analysis (see Table 3) indicated that the difference in MPA_D [β = −0.310, *p* < 0.01, 95% CI (−0.500, −0.120)] and SPA_T [β = 0.160, *p* < 0.01, 95% CI (0.030, 0.290)] was significantly associated with differences in individuals’ DS. Among the control variables, gender [β = −1.050, *p* < 0.01, 95% CI (−1.740, −0.360)], sleep latency [β = 0.07, *p* < 0.001, 95% CI (0.05, 0.09)], alcohol consumption [β = 0.38, *p* < 0.05, 95% CI (0.08, 0.68)], and smoking status [β = −0.840, *p* < 0.01, 95% CI (−1.580, −0.090)] were also significantly related with differences in the participants’ DS. The effect size of the Bayesian regression model was adequate, and 17.1% of the variance in differences in DS was predicted by the proposed model. We further conducted a multi-group moderation analysis to examine if the relationships between differences in seven PA measures and differences in DS varied across the three age clusters.

**TABLE 3:** The results of Bayesian regression analysis of changes in physical activity on depressive symptoms.

Factors	Parameter	Δ in DS (Adjusted *R*^2^ = 17.1%)			
		Posterior		95% CI	
		Mean	Variance	Lower bound	Upper bound
	Intercept	5.72**	4.34	1.64	9.80
Physical Activities	Δ_VPA_D	−0.13	0.01	−0.32	0.06
	Δ_VPA_T	0.03	0.01	−0.12	0.19
	Δ_MPA_D	−0.31**	0.01	−0.50	−0.12
	Δ_MPA_T	0.03	0.01	−0.14	0.21
	Δ_WPA_D	−0.02	0.01	−0.19	0.14
	Δ_WPA_T	−0.10	0.01	−0.27	0.07
	Δ_SPA_T	0.16*	0.00	0.03	0.29
Personalcharacteristics	Young	−1.25**	0.21	−2.16	−0.35
	Middle-aged	−0.88*	0.20	−1.75	−0.001
	Caucasian	−2.06	3.63	−5.79	1.68
	African-American	−1.88	3.99	−5.80	2.03
	Hispanic	−3.58	3.91	−7.45	0.30
	Asian	−2.52	3.98	−6.43	1.39
	Native American	−2.01	4.08	−5.97	1.95
	Gender_Male	−1.05**	0.12	−1.74	−0.36
	Education	−0.20	0.03	−0.51	0.11
	Income	−0.02	0.02	−0.30	0.27
	BMI	0.01	0.00	−0.05	0.06
Non-physical-activity health behaviors	Δ_SQ1_H	−0.15	0.01	−0.34	0.04
	Δ_SQ2_M	0.07***	0.00	0.05	0.09
	Δ_OSQ	−0.31	0.04	−0.71	0.09
	Δ_DQ_H	0.00	0.00	−0.06	0.05
	Δ_DQ_UH	0.02	0.00	−0.03	0.07
	Δ_Alcohol	0.38*	0.02	0.08	0.68
	Smoke_Yes	−0.84*	0.15	−1.58	−0.09

*Dependent Variable: Δ_CESD_Total between January and May; Baseline of age_group = Old; Baseline of race = Two or more ethnicities. Baseline groups were coded as zero in newly created dummy variables.*

*CI, Bayesian credible intervals; D, number of days per week; DQ, dietary quality; DS, depressive symptoms; MPA, moderate intensity physical activity; OSQ, overall sleep quality; SPA, sitting; SQ, sleep quality; T, total time in hours; VPA, vigorous intensity physical activity; WPA, walking.*

*p < 0.05; **p < 0.01; and ***p < 0.001.

Although young [β = −1.25, *p* < 0.01, 95% CI (−2.16, −0.35)] and middle-aged cohorts [β = −0.88, *p* < 0.05, 95% CI (−1.75, −0.001)] experienced significantly fewer differences in DS compared to their counterparts in the older group (see Table 2 and Figure 2), we found no significant heterogeneity effects in the relationships of interest between the age groups. For instance, despite increases in sitting time being significantly associated with a deteriorating difference in DS across all samples, the strength of the relationship was comparable between young and middle-aged groups [β*_*diff*_* = 0.009, *p* = 0.928, 95% CI (−0.010, 0.055)], young and older adult segments [β*_*diff*_* = −0.099, *p* = 0.305, 95% CI (−0.010, 0.093)], and middle-aged and older clusters [β*_*diff*_* = −0.109, *p* = 0.329, 95% CI (−0.083, 0.083)]. Similarly, there was no significant difference between differences of other measures of PA and differences in DS between young, middle-aged, and older adults.

### Robustness Checks With Machine Learning

**FIGURE 3:**
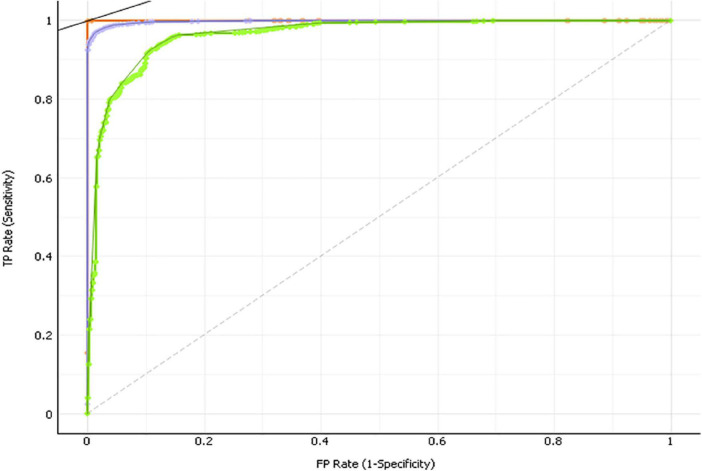
Results of feature importance and ROC curves reflecting the predictive accuracy using machine learning.

**FIGURE 4:**
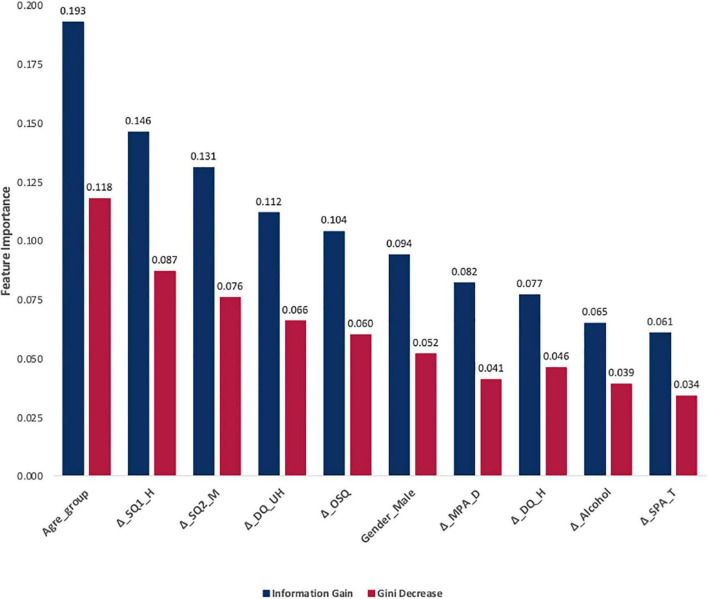
Rankings of feature importance of the included behavioral and demographic correlates. Navy blue represents the ranking based on feature importance scores using Information Gain values. Glory red denotes the ranking based on feature importance scores based on Gini Decrease values.

The visualizations of ROC curves corroborated with overall prediction accuracy based on confusion matrices indicated that both RF and SVM were able to significantly minimize the misclassification rate (Podsakoff et al., 2003). The overall classification accuracy (CA) was computed by using the total number of people correctly classified as depressed and undepressed, divided by a sum of four possible prediction outcomes (e.g., true depressed, true undepressed, false depressed, and false undepressed). In Figure 3, the orange curve refers to RF (CA = 99.50%), the purple curve represents support vector machines (SVM) (CA = 97.4%), and the green curve reflects Naïve Bayes (NB) (CA = 91.3%). Finally, Figure 4 presents the rankings of feature importance of the included behavioral and demographic correlates. The ranking of GD and IG indicated that Age_group, Δ_SQ1_H, Δ_SQ2_M, Δ_DQ_UH, Δ_OSQ, Δ_MPA_D, Δ_DQ_H, Δ_Alcohol, and Δ_SPA_T were consistently identified as the most critical behavioral and demographic attributes to positively impact the prediction results of classification, which were mostly consistent with the findings from the Bayesian regression analysis, thereby confirming the robustness of our results (see Figures 3, 4).

## Discussion

Several scholars have warned that social isolation guidelines from the COVID-19 pandemic can increase physical inactivity, which negatively impacts physical health (i.e., the immune system, respiratory system, cardiovascular system, and musculoskeletal system) (Woods et al., 2020) and mental health (Pecanha et al., 2020). The primary aim of this study was to explore potential changes in levels of PA, NHB, and DS; and how changes in PA were associated with changes in one’s DS among three different populations—young adults, middle-aged adults, and older adults—in the United States before (January of 2020) and after (May of 2020) the initiation of COVID-19-related social isolation guidelines.

The findings indicated that only the older adults’ VPA frequency was significantly increased while all the other VPA, MPA, and WPA levels of the young, middle-aged, and older respondents were not significantly changed. On the contrary, significant increases in the levels of sedentary behavior (sitting time) were found, especially among the young and older respondents. These findings diverge from the results of previous studies. For instance, Lopez-Bueno et al. (2020) noticed weekly PA levels reduced by 20% during the first week of COVID-19 lockdown in Spanish adults. Similarly, Yamada et al. (2020) found a significant decrease in total PA time among community-dwelling older adults in Japan. However, the inconsistency may be due to the fact that their study examined PA levels immediately after the confinement, which were in March of 2020 and April of 2020 respectively, whereas the current research examined PA levels in May of 2020. Possibly, the reduced PA levels reverted to the baseline level as the lockdown lasted more than a month.

The World Health Organization (WHO) recommends at least 150 min of MPA or 75 min of VPA per week, or a combination of both for adults, with a duration of 300 min of MPA for additional health benefits [World Health Organization (WHO), 2018]. It should be noted that, as of May of 2020, the study’s participants reported an average of 466 min of VPA and 502 min of MPA per week. The older participants noted the lowest levels with an average of 178 min of VPA and 267 min of MPA per week, which still is higher than the WHO recommendations. This finding is somewhat consistent with Meyer et al. (2020) in that their United States samples also reported being at least sufficiently active across all three age groups in April of 2020. Unlike the PA levels, notably, the respondents—especially, young adults and older adults—experienced higher levels of sitting time during the lockdown. These findings are consistent with previous commentaries (e.g., Hall et al., 2020; Woods et al., 2020). Yet, even though there were few empirical studies on the changes of PA levels, no empirical studies on the changes of sedentary behavior have been published to date.

Despite the fact that several previous studies predicted a significant decrease in healthy diet and increase in unhealthy diet (Mattioli et al., 2020b), we found significant increases in healthy food consumption across all three groups, whereas only young adults had a significant increase in unhealthy food consumption. The older adults even experienced a significant decrease in unhealthy food consumption. These relatively promising results may be due to the relatively high socioeconomic status of respondents. Moreover, since older COVID-19 patients tended to experience more significant health issues than their younger counterparts, older participants in the present study may have been more motivated to adopt positive lifestyle changes. The results of alcohol consumption also contradicted many previously published expectations (Mattioli et al., 2020a) since young respondents in our study reported significant decreases in alcohol consumption. Similarly, our finding of no changes in smoking levels and relapsing was also not congruent predicted behaviors (Patwardhan, 2020). Though respondents in the present study reported longer hours of sleep, they also noted more minutes were required to fall asleep after the lockdown, which was consistent with the results of prior research (Pinto et al., 2020; Wright et al., 2020).

Consistent with expectations (Purtle, 2020; Zandifar and Badrfam, 2020), the middle-aged and older participants had a significantly higher level of DS after the lockdown. Compared to the younger and middle-aged groups, particularly, the older respondents had significantly higher levels of DS after the lockdown as Figure 2 presented. This may seem alarming, but it should be noted that the baseline of the older participants’ DS was well below the threshold (CESD_Total = 5.01), whereas middle-aged (CESD_Total = 13.19) and younger adults (CESD_Total = 15.15) showed mild levels of DS in January of 2020. In other words, even though the older adults were experiencing more negative changes in DS, the group still had the lowest level of DS compared to the other counterparts in May of 2020. The findings support the previous studies on the relationship between aging and happiness in that happiness tends to decline from early adulthood to middle adulthood and turns back up as people age showing a U-shaped relationship (e.g., Frijters and Beatton, 2012; Graham and Ruiz Pozuelo, 2017).

After controlling NHB and personal characteristics, regardless of the age differences, the changes in frequency of MPA and sitting time were significantly associated with the changes in DS. Less frequent MPA and increased sitting time predicted a higher level of DS, a result consistent with previous studies (Mattioli et al., 2020a). In particular, the frequency of MPA, rather than the duration, mattered for DS. Considering that DS levels are assessed daily, more days with MPA may better combat DS than longer hours of doing MPA. Therefore, instead of combined minutes of PA, highlighting minimum days of PA may be even more important for mental health outcomes such as DS.

According to Rajkumar (2020), the research topics of published articles on the influence of COVID-19 on mental health have been very limited, including only observational studies on mental health symptoms in particular populations, commentary, and correspondence addressing the psychological impact of COVID-19 on the general populations, healthcare workers, and vulnerable populations. Consequently, there has been no intervention-based study to develop more therapeutic strategies and programs *via* active lifestyle to address mental health issues resulting from COVID-19, a necessary prerequisite of one’s individual psychological well-being. For future intervention-based studies, it may be important to differentiate PA effects on mental health depending on the different levels of DS and different domains of PA (e.g., leisure-time PA, commuting PA, work-related PA; Appelqvist-Schmidlechner et al., 2020). In fact, few studies examined the relationship between exercise intensity and relief of depression through endorphin secretion. With moderate levels of depression, one study showed that only moderate- and high-intensity exercise could relieve depression levels of that magnitude (Balchin et al., 2016).

As social distancing policies continue due to the pandemic, various types of interventions should be newly developed to embrace the changing landscapes of social spaces. In order to provide resources in an easily accessible manner without any increase in infection risk, we need to consider technology-driven interventions for promoting an active lifestyle. Because digital technology offers more accessible resources, for instance, mobile health apps have been attractive to various populations due to their flexibility and ease of use (Anthes, 2016). To date, several studies have examined the effectiveness of mobile apps and wearable devices for PA promotion, but most studies have measured PA levels with simple step counts (Brickwood et al., 2019; Feter et al., 2019; Yerrakalva et al., 2019). Considering the results of the present study showed differing influences of PA on mental health depending on different levels and domains, more intervention studies should be conducted with an assessment that differentiates PA levels in the future.

Because the current study was an observational and exploratory study, employing the Bayesian approach provides several advantages compared to the conventional frequentist inferential statistics, which relies on the principle of long-run frequency and standard null-hypothesis significance-testing procedure (Trafimow, 2003). First, the Bayesian approach is suitable to integrate informative priors (e.g., individuals’ baseline health conditions prior to the outbreak of the COVID-19 pandemic in January) into empirical probabilistic models (Gelman, 2004). Moreover, this approach does not require the asymptotic assumptions (i.e., multi-normality based on large samples) and is less computationally demanding (Kaplan, 2014). Lastly, the conclusions of Bayesian analyses are less sensitive to outliers and missing data, more robust to handle small sample sizes, and easier to interpret with the intuitive predictive posterior credible intervals (Rupp et al., 2004). Based on the results of exploratory studies like the current research, future studies from a deductive approach to examine the unintended long-term consequences of the COVID-19 pandemic by leveraging cumulative longitudinal data are needed.

### Limitations and Future Implications

Our study included a number of limitations that have implications for future research. First, our convenience sample of United States residents featured a relatively healthy population with high levels of PA, low levels of DS, and an average or higher socioeconomic status. Accordingly, our results should not be generalized to dissimilar populations. Second, we included sociodemographic information, BMI, and NHB as covariates, but there may be other major variables that may significantly influence one’s PA level and DS. For instance, in the field of social epidemiology (i.e., an academic field interested in the impacts of social factors on health and disease distribution in populations; El-Sayed et al., 2012), previous studies found that online social networking may impact the spread of non-infectious conditions such as smoking, alcohol consumption, tobacco use, or physical (in)activity (Maher et al., 2014). These factors related to social media consumption and website usage may need to be considered in future PA and DS studies. Additionally, we asked participants to recall their behavior 5 months ago. Considering that COVID-19 is a historical and impactful global event for every person, it is reasonable to expect more people would remember their behaviors and moods around the event. Nevertheless, recall bias can be a concern for this type of cross-sectional data. Lastly, high levels of PA among our respondents may be due to the impact of social desirability on self-report PA questionnaire, a possibility that cannot entirely be ruled out (Crutzen and Göritz, 2011; Brenner and DeLamater, 2014).

Finally, several studies have noted that existing health inequalities may be amplified as a consequence of the COVID-19 lockdown. For example, Lopez-Bueno et al. (2020) found a stronger effect of PA reduction ingroups with lower education. Considering that the majority of the present sample tended to have relatively high socioeconomic status (e.g., higher education, higher employment rate), further studies with underrepresented populations should be conducted to grasp a more complete picture of the effect of the COVID-19 lockdown on the public.

## Data Availability Statement

The original contributions presented in the study are included in the article/supplementary material, further inquiries can be directed to the corresponding author.

## Ethics Statement

The studies involving human participants were reviewed and approved by Florida State University. The patients/participants provided their written informed consent to participate in this study.

## Author Contributions

AK, JD, and DA contributed to the conception and design of the study and wrote sections of the manuscript. AK organized the data collection. JD performed the statistical analyses. All authors contributed to manuscript revision, read, and approved the submitted version.

## Conflict of Interest

The authors declare that the research was conducted in the absence of any commercial or financial relationships that could be construed as a potential conflict of interest.

## Publisher’s Note

All claims expressed in this article are solely those of the authors and do not necessarily represent those of their affiliated organizations, or those of the publisher, the editors and the reviewers. Any product that may be evaluated in this article, or claim that may be made by its manufacturer, is not guaranteed or endorsed by the publisher.
